# Evaluation of survivin splice variants in pituitary tumors

**DOI:** 10.1007/s11102-014-0590-9

**Published:** 2014-08-09

**Authors:** Joanna Waligórska-Stachura, Mirosław Andrusiewicz, Nadia Sawicka-Gutaj, Marta Kubiczak, Anna Jankowska, Włodzimierz Liebert, Agata Czarnywojtek, Ryszard Waśko, Al Ricardo Blanco-Gangoo, Marek Ruchała

**Affiliations:** 1 Department of Endocrinology, Metabolism and Internal Diseases, Poznan University of Medical Sciences, Przybyszewski Street 49, 60-355 Poznań, Poland; 2 Department of Cell Biology, Poznan University of Medical Sciences, Rokietnicka St. 5d, 60-806 Poznań, Poland; 3 Department of Neurosurgery and Neurotraumatology, Poznan University of Medical Sciences, Przybyszewski St. 49, 60-355 Poznań, Poland

**Keywords:** Survivin, Survivin splice variants, RT-qPCR, Pituitary tumors

## Abstract

**Purpose:**

Survivin is an apoptosis inhibitor, expressed in almost all types of human malignancies, but rarely in differentiated normal tissues. Recently, survivin gene splice variants with different anti-apoptotic activities have been reported. The current study was undertaken to examine the expression of survivin and its splice variants ∆Ex3 and 2β in pituitary tumors, and to correlate the amount of particular transcripts with clinical staging in pituitary adenomas. Quantitative detection of survivin and its splice variants ∆Ex3 and 2β transcripts in non-cancerous pituitary tissues (n = 12) and different types of pituitary tumor (n = 50).

**Methods:**

Samples were collected from 50 pituitary tumors including 26 non-functional tumors, 21 GH-secreting tumors, 2 PRL-secreting tumors and 1 ACTH-secreting tumor. 12 normal pituitary glands received after autopsy served as a control of the study. 29 thyroid cancers tissues were used as a positive control. The RT-qPCR with TaqMan hydrolysis probes were used to determine the expression of analyzed splice variants of survivin.

**Results:**

The obtained data showed that both survivin and its splice variants were expressed in different types of pituitary adenoma as well as in normal pituitary tissue. However, the level of its expression was similar in all studied cases. Patient age negatively correlated with tumor invasiveness. Moreover, our study showed a tendency for negative correlation between patient age and tumor diameter.

**Conclusions:**

No significant differences between survivin and its splice variants ∆Ex3 and 2β expression in pituitary tumors and in normal pituitary glands as well as in invasive and in non-invasive tumors were found, suggesting that survivin does not play a significant role in pituitary tumorigenesis.

## Introduction

Pituitary adenomas are the most common tumors in the central nervous system and are thought to be monoclonal in origin [1].

Little is known about the pathogenesis of pituitary neoplasia. Previous studies suggested that pituitary tumorigenesis may be promoted by molecular events such as: increased transforming gene expression, silencing of tumor suppressor genes (TSGs), pituitary and hypothalamic hormonal dysregulation in addition to environmental or other mutagenic stimuli [2–5]. As was reported by Melmed’s group, pituitary tumor transforming gene (PTTG) is a molecular marker for invasiveness in hormone-secreting pituitary tumors. The abundant expression of PTTG in pituitary adenomas suggests that it plays a major role in pituitary tumorigenesis and invasiveness [6–8].

In spite of the fact that pituitary tumors are mostly benign adenomas, some of these tumors invade tissues outside of the pituitary gland. It makes it difficult to achieve complete removal at surgery and leads to a strong tendency to recur. It was a reason why we wanted to determine if invasive pituitary tumors express higher levels of survivin and its splice variants and whether the level of survivin expression differs between different types of pituitary tumor.

Many studies also linked pituitary tumors with survivin [9–12]. It is the smallest member of the IAP (inhibitor of apoptosis protein) family controlling chromosome compaction, mitotic spindle formation and microtubule dynamics. At the molecular level, survivin is a multifunctional protein, which not only plays a central role in cell division, but also in suppressing apoptosis and enhancing angiogenesis [13–15].

Survivin has been shown to be expressed only during mitosis. Its expression increases in the G2/M phase and decreases rapidly in G1. Its expression is regulated by a number of factors [9, 10]. In addition to the full-length transcript, four alternative splice variants of the survivin gene product have been described: ∆Ex3, 3β, 2β and 2α [4]. Survivin splice isoforms play different roles in the cell-cycle. Survivin ∆Ex3 was shown to confer anti-apoptotic activities, while survivin 2β antagonizes with anti-apoptotic properties. Many reports suggest that ∆Ex3 and 3β are cytoprotective, while 2β and 2α are pro-apoptotic. Survivin ∆Ex3 has also been associated with higher tumor staging, increased tumor aggressiveness and poor prognosis especially in breast, gastric and thyroid cancers [15–21].

Until now, survivin overexpression was observed in a variety of cancers. Survivin overexpression was found in 96 % of lung cancer specimens, 100 % of colon adenocarcinomas, 71 % of prostate adenocarcinomas, 80 % of glioblastomas and 100 % of laryngeal carcinomas [22–33]. Survivin synthesis correlates with an unfavorable clinical outcome. Recently the prognostic value of its different splice variants has been considered [18, 32, 34].

Data concerning the survivin expression in pituitary tumors and its involvement in pituitary tumorigenesis is contradictory. In this study, we assessed the expression of survivin and splice variants ∆Ex3 and 2β in different types of pituitary tumor and correlated their levels with clinical data including tumor invasiveness, size, functionality and patient age. Our goal was to evaluate whether survivin splice variants are involved in pituitary tumorigenesis and if it could serve as a predictive marker in the clinical outcomes of pituitary tumors.

## Materials and methods

### Patient demographic data and tumor size

Patients hospitalized in the Department of Neurosurgery and Neurotraumatology, University of Medical Sciences in Poznań, were recruited for the purpose of this study. The research was approved by the ethics review board of Poznań University of Medical Sciences and all participants provided written informed consent.

The average age at diagnosis was 53 (±14) years with 33 female and 17 male patients. Information regarding tumor size was obtained after reviewing pre-operative MRI scans. The tumor sizes ranged between 13 and 55 mm at the largest diameter. Tumors with a diameter above 2.5 cm were categorised as large, and those below 2.5 cm as small. The examined pituitary tumor group consisted of 35 large tumors and 14 small tumors. According to both pituitary MRI scans and intraoperative neurosurgical opinion, pituitary tumors were divided into 36 invasive and 14 non-invasive tumors. Invasion was defined as an infiltration and often destruction of parasellar tissues, including the dura, bone, cavernous venous sinuses, cranial nerves, paranasal sinuses, subarachnoid space, and leptomeninges. Division into invasive and non-invasive pituitary tumors was made using radiological evidence of invasion on magnetic resonance imaging or/and by neurosurgeon at surgery (intraoperative inspection of the sellar walls and parasellar tissues).

### Tumor specimens

Pituitary adenomas were obtained by transsphenoidal surgery from 50 patients and biochemically and histologically classified into non-functional (n = 26) and functional (n = 24) tumors. The latter group consists of 21 GH-secreting tumors, 2 PRL-secreting tumors and 1 ACTH-secreting tumor. Patients with acromegaly were treated with somatostatin analogues 3–6 months prior to surgery. A negative control consisted of 12 normal pituitary glands without cancerous changes, obtained post-mortem. Tissue samples obtained from 29 patients who had undergone thyroid removal and with pathological confirmation of thyroid cancer, were used as a positive control.

Resected tissues were immediately stored in RNAs protective medium—RNALater (Sigma Aldrich) for following mRNA isolation. RNA extraction and reverse transcription were followed by quantitative PCR (RT-qPCR).

### RNA extraction and analysis

Total cellular RNA was extracted according to the TriPure Isolation Reagent manufacturer’s protocol (Roche Diagnostic GmbH, Mannheim, Germany). The concentration and the quality of total RNA were determined spectrophotometrically (NanoDrop ND-1000 spectrophotometer; Thermo Fisher Scientific, Waltham, MA) and its integrity was electrophoretically confirmed on denaturizing agarose gel, throughout visible 18S and 28S rRNA bands.

### Reverse transcription and quantitative PCR (RT-qPCR)

Complementary DNA (cDNA) was synthesized according to the manufacture’s reverse transcriptase protocol using: 1 ng/μl of total RNA, 5 pmol/μl universal oligo(d)T_10_ primer, 10U/μl Transcriptor Reverse Transcriptase, 1× Expand Reverse Transcriptase Buffer, 10U/μl RNasin RNase inhibitor and 1 pmol/μl of each dNTP (deoxynucleoside triphosphate) (Roche Diagnostic GmbH). As a negative ‘no template control’ (NTC), a sample in which reverse transcriptase was replaced with water in the reaction mixture was used.

To assess the total expression level of BIRC5 [NCBI: NM_001168], BIRC5-ΔEx3 [NCBI: NM_001012270.1], BIRC5-2B [NCBI: NM_001012271.1] and HPRT reference gene [Human HPRT Gene Assay Cat. No. 05 046 157 001 (Roche Diagnostics)] real-time PCR with sequence specific primers (Table 1) was applied. TaqMan hydrolysis probes and LightCycler^®^ TaqMan^®^ Master Kit were used. TaqMan hydrolysis probes for the examined genes (GOI, gene of interest) were designed using ProbeFinder Software (version 2.50) (21, 22) and they were purchased from the collection of Universal Probe Library (UPL) (Roche Diagnostics). Each reaction was conducted in triplicate using independently synthesized cDNA.

**Table 1:** Primers and the TaqMan hydrolysis probes used in this study

Gene	TaqMan probe No	Forward primer 5′ → 3′	Reverse primer 5′ → 3′	Amplicon
Total BIRC5	#36 (Cat. No. 04687949001)	gcccagtgtttcttctgctt	aaccggacgaatgcttttta	88 bp
BIRC5-ΔEx3	#36 (Cat. No. 04687949001)	cagtgtttcttctgcttcaagg	cttattgttggtttcctttgcat	77 bp
BIRC5-2B	#36 (Cat. No. 04687949001)	tctgcttcaaggagctgga	aaagtgctggtattacaggcgta	88 bp
HPRT	Human HPRT Gene Assay, Cat. No. 05 046 157 001 (Roche Diagnostics)			

The RT-qPCR reaction was carried out in a reaction volume of 20 μl. The reactions were conducted according the LightCycler^®^ TaqMan^®^ Master manufacture’s protocol (Roche Diagnostic GmbH). The reaction mixture and the thermal profile were shown in Tables 2 and 3 respectively. The qPCR reaction was performed in triplicate using a LightCycler^®^ 2.0 instrument (Roche Diagnostic GmbH) with independently synthesized cDNA. The fluorescence emission was measured at the 530 nm channel for GOI genes and 560 nm UPL reference gene.

**Table 2:** qPCR reaction mixture compounds

Component	Final concentration
cDNA	5 μl
Forward and reverse primer’s mix	0.5 pmol/μl
TaqMan hydrolisis probe	0.1 μM
LightCycler FastStart TaqMan Reaction Mix	1×
PCR grade water	To 20 μl

**Table 3:** qPCR thermal profile

Cycles	Analysis mode		Target temperature, hold time	Acqusition mode
1	Pre-incubation		95 °C, 10 min	None
45	Quantification	Denaturation	95 °C, 10 s	None
		annealing, extension	60 °C, 20 s	None
		Fluorescence data acquisition	72 °C, 1 s	Single
1	Cooling		40 °C, 30 s	None

Standard curves were constructed for each gene separately with decimal dilution of the cDNA library constructed from OVCAR3 cell line (ATCC^®^), starting from undiluted cDNA up to a dilution of 10^−5^ to calculate the PCR reactions efficiencies. The standard curves cycling reactions were conducted in triplicate for each gene, and the efficiency values were obtained from the standard curves using the efficiency correction. Each of the reaction sets involved NTC control. Since contamination was not observed, the Uracil-DNA glycosylase incubation step was omitted. After the standard curve cycling reactions, a linear fit was performed using LightCycler Data Analysis Software. Cp-values were plotted against log concentration. The slope of regression was converted into PCR efficiency (E = 10^−1/slope^) and those values were stored as the standard curve and used for subsequent reaction analysis.

### Data collection

PCR results were assembled using the LightCycler^®^ Data Analysis (LCDA) Software version 4.0.5.415 dedicated for the LightCycler^®^ 2.0 instrument. Baseline and threshold values were automatically set by the software. The number of PCR cycles required to reach fluorescence over the background was defined as the crossing point (Cp). Each sample was analyzed in triplicate, and the average Cp value was calculated. After normalization of results using the HPRT reference gene and efficiency correction with standard curves of each gene, the concentration value for the study genes was calculated. The relative expression of the analyzed genes normalized with the HPRT gene was shown as concentration ratios (Cr). The obtained data was used for statistical analyses.

### Statistical analysis

Statistical analyses were performed with MedCalc version 12.1.3.0 (MedCalc Software, Mariakerke, Belgium). Normality was analyzed by D’Agostino-Pearson test. Data did not follow normal distribution. Therefore, comparisons of the analyzed parameters between two groups were performed with the Mann–Whitney test, and the nonparametric Spearman’s rank-correlation test was used to analyze the relationships between the level of survivin expression, tumor diameter, invasiveness and patients’ age and gender. The results were considered to be statistically significant if the *P* value was lower than 0.05.

## Results

**Fig. 1:**
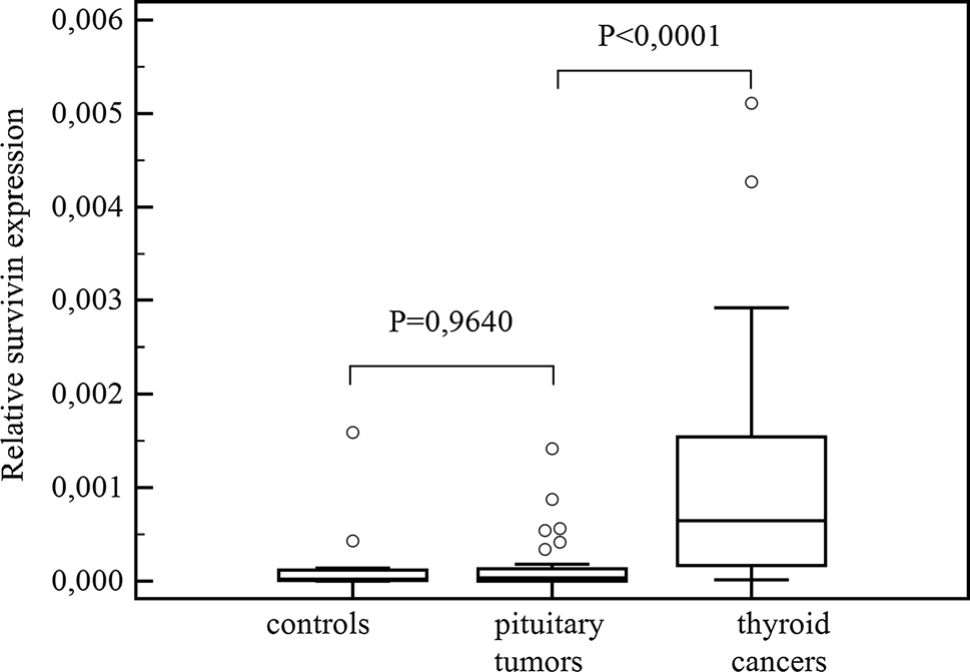
Comparisons of survivin expression in pituitary tumors, in healthy controls and in thyroid cancers. *Central box* represents the values from the lower to upper quartile (25th to 75th percentile). The *middle line* represents the median. The *thin vertical lines* extending up or down from the *boxes* to *horizontal lines* (so-called whiskers) extend to a multiple of 1.5× the distance of the upper and lower quartile, respectively. Outliers are any values beyond the whiskers

**Fig. 2:**
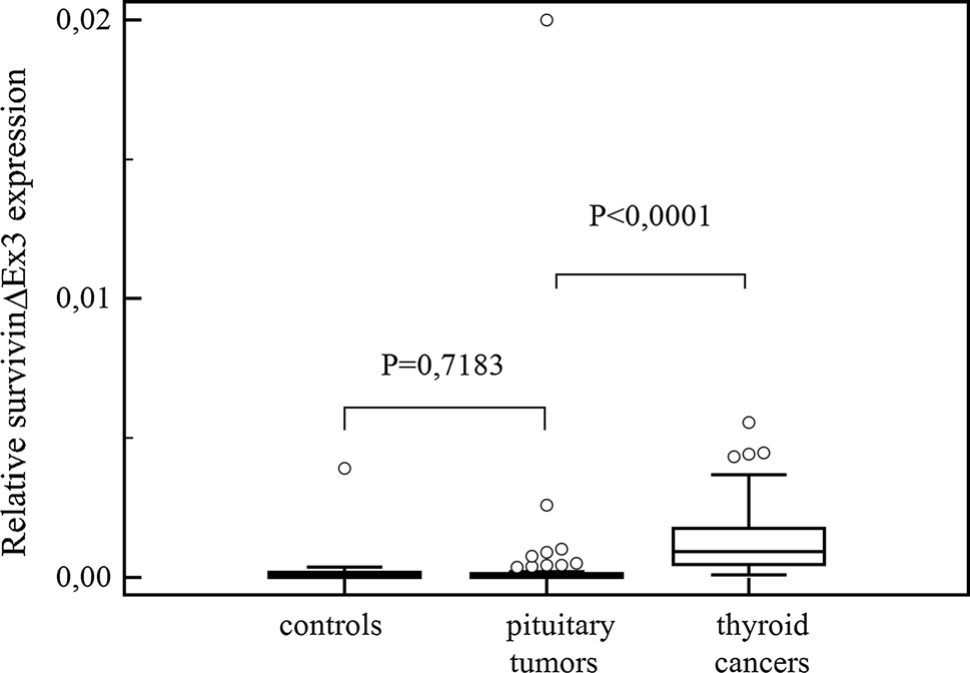
Comparisons of survivin ∆Ex3 expression in pituitary tumors, in healthy controls and in thyroid cancers. *Central box* represents the values from the lower to upper quartile (25th to 75th percentile). The *middle line* represents the median. The *thin vertical lines* extending up or down from the *boxes* to *horizontal lines* (so-called whiskers) extend to a multiple of 1.5× the distance of the upper and lower quartile, respectively. Outliers are any values beyond the whiskers

**Fig. 3:**
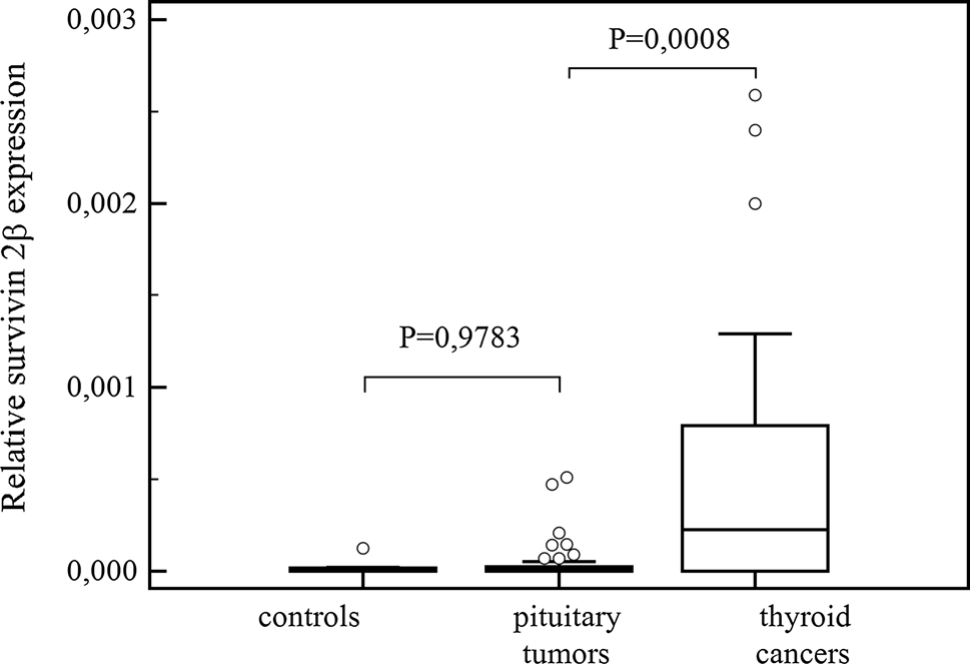
Comparisons of survivin 2β expression in pituitary tumors, in healthy controls and in thyroid cancers. *Central box* represents the values from the lower to upper quartile (25th to 75th percentile). The *middle line* represents the median. The *thin vertical lines* extending up or down from the *boxes* to *horizontal lines* (so-called whiskers) extend to a multiple of 1.5× the distance of the upper and lower quartile, respectively. Outliers are any values beyond the whiskers

**Fig. 4:**
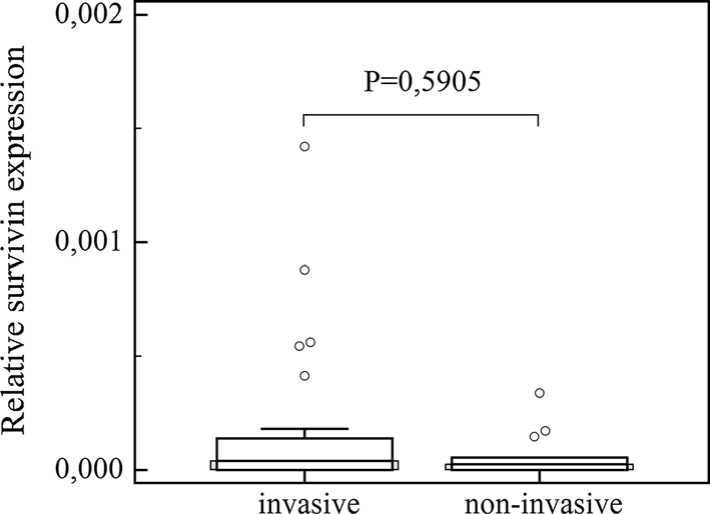
Comparison of survivin expression in invasive and non-invasive pituitary tumors. *Central box* represents the values from the lower to upper quartile (25th to 75th percentile). The *middle line* represents the median. The *thin vertical lines* extending up or down from the *boxes* to *horizontal lines* (so-called whiskers) extend to a multiple of 1.5× the distance of the upper and lower quartile, respectively. Outliers are any values beyond the whiskers

**Fig. 5:**
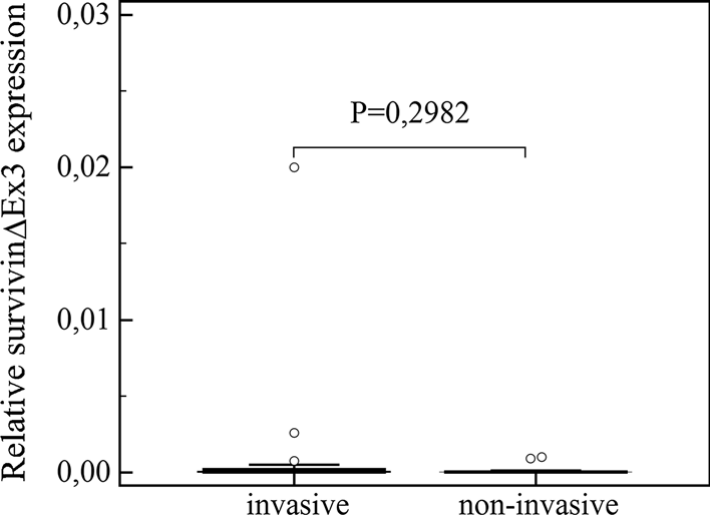
Comparison of survivin ∆Ex3 expression in invasive and non-invasive pituitary tumors. Outliers are shown as *dots*

**Fig. 6:**
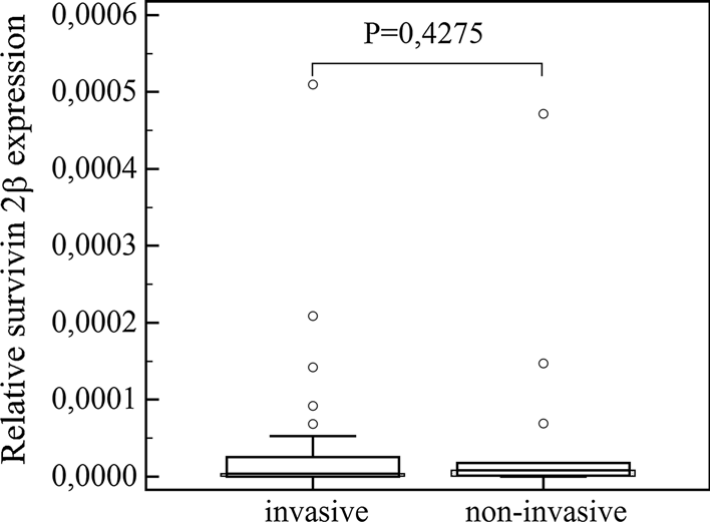
Comparison of survivin 2β expression in invasive and non-invasive pituitary tumors. *Central box* represents the values from the lower to upper quartile (25th to 75th percentile). The *middle line* represents the median. The *thin vertical lines* extending up or down from the *boxes* to *horizontal lines* (so-called whiskers) extend to a multiple of 1.5× the distance of the upper and lower quartile, respectively. Outliers are any values beyond the whiskers

The obtained data (presented in Figs. 1, 2, 3) showed that both survivin and its splice variants were expressed in different types of pituitary adenoma as well as in normal pituitary tissue. Furthermore, the level of its expression was similar in all studied cases (survivin, *P* = 0.9640; ∆Ex3, *P* = 0.7183; and 2β, *P* = 0.9783). A lack of statistically important changes in the level of analyzed transcripts was shown in the case of invasive and non-invasive pituitary tumors (Figs. 4, 5, 6: survivin, *P* = 0.5905, ∆Ex3, *P* = 0.08620, 2β, *P* = 0.0818).

We found no difference in survivin variant expression between large and small pituitary tumors (survivin, *P* = 0.5985; ∆Ex3, *P* = 0.6935; 2β, *P* = 0.6303) nor between functional and non-functional ones (survivin, *P* = 0.6181; ∆Ex3, *P* = 0.3334; 2β, *P* = 0.4878).

Also, the comparison of survivin and splice variant expression in GH-secreting tumors preoperatively treated with somatostatin analogues with other pituitary tumors, revealed that their expression is similar in all studied cases (survivin *P* = 0.5397; ∆Ex3 *P* = 0.1851; 2β, *P* = 0.2752).

A comparison of the variation in survivin expression between pituitary tumors and positive control-thyroid cancers revealed a significantly higher expression of survivin (*P* < 0.0001), and its variants: ∆Ex3, *P* < 0.0001 and 2β, *P* = 0.0008 in the thyroid cancer group (Figs. 1, 2, 3).

Patient age negatively correlated with tumor invasiveness (*P* = 0.0404; r = −0.339). Moreover, our study showed a tendency for negative correlation between patient age and tumor diameter (*P* = 0.0627; r = −0.301).

There was no significant correlation between survivin variant expression and gender.

## Discussion

Previous publications concerning brain tumors indicated that quantifying the levels of survivin and its splice variants is useful for predicting the cell biological malignancy of gliomas, independent of their pathological features [9, 10]. Therefore, in this present study, we decided to examine wild survivin as well as the expression of its splice variants ∆Ex3 and 2β in pituitary tissues, and also to determine whether the levels would correlate with pituitary tumor invasiveness, size, functionality, patient sex and age.

Our study, including 50 different pituitary tumor samples and 12 pituitary samples without cancerous changes, demonstrated the presence of survivin and its splice variants transcripts in both normal pituitary tissues and in pituitary tumors. The level of survivin splice variant expression in pituitary adenomas was similar to those found in normal pituitary. There was no correlation between their expression in invasive tumors and non-invasive ones.

A limited number of articles regarding survivin expression and its importance in pituitary tumors are available but they present contradictory data. Previously, Formosa’s group examined survivin presence in 47 pituitary adenomas using immunohistochemistry and showed that survivin expression was extremely low in tumors and absent in normal pituitary tissues. Survivin expression was present in less than 1 % of tumor cells [11].

In another study, high survivin expression in invasive pituitary tumors was showed by immunohistochemistry. In comparison with non-invasive adenomas, staining intensity was observed to be less intense in those tumors [12].

In our previous research, we showed that survivin was expressed at a higher level in pituitary tumors, but was also present in normal pituitary tissues. Immunostaining localized survivin mainly within cell nuclei and revealed the coexpression of survivin with PCNA (proliferating cell nuclear antigen), especially in invasive tumors [9]. However, the former study included a much smaller group of pituitary tumors.

Currently, we evaluated the survivin and its splice variants in functional and non-functional pituitary tumors. We compared the level of survivin expression in acromegalic patients treated with somatostatin analogues, with other functional and non-functional tumors. We found no difference in the amount of survivin and its variants between these tumors. Expression levels of survivin and variants ∆Ex3 and 2β were similar in patients with functional tumors requiring octreotide treatment and in patients with non-functioning tumors.

The results demonstrated no correlation between survivin expression and the patients’ clinical status. They revealed a link between pituitary tumor invasiveness and patient age. Moreover, a *tendency* for larger tumors in younger patients was observed.

## Conclusion

The performed study revealed a comparable levels of survivin expression and its splice variants in pituitary tumors and in normal pituitary. Also, the results of our study did not show a significant difference in survivin expression between invasive and non-invasive pituitary tumors, as well as functional and non-functional adenomas. The comparison of survivin expression in GH-secreting tumors preoperatively treated with somatostatin analogues with other pituitary tumors, revealed a similar survivin level of expression in all cases.

Moreover, we found a significantly lower expression of survivin splice variants in pituitary tumors than in thyroid cancers.

Further investigations concerning the regulatory mechanisms of survivin expression and function in normal and cancerous cells will help to elucidate survivin’s biology and will help to understand endocrine tumor development.
